# Clinicians’ Perspectives on Barriers and Opportunities in Implementing DETAILD for Enhancing the Care of Patients Living with Behavioral and Psychological Symptoms of Dementia and Their Caregivers

**DOI:** 10.1002/alz70860_098682

**Published:** 2025-12-23

**Authors:** Molly Schroeder, Tamara J. LeCaire, Tammi Albrecht, Jonathan A Stone, Uriel Paniagua, Sylvia Peng, Stephanie Houston, Sarina Schrager, Cynthia M. Carlsson, Art Walaszek

**Affiliations:** ^1^ Wisconsin Alzheimer's Institute, University of Wisconsin School of Medicine and Public Health, Madison, WI, USA; ^2^ University of Wisconsin‐Madison School of Medicine and Public Health, Madison, WI, USA; ^3^ UW Madison School of Medicine and Public Health, Madison, WI, USA; ^4^ UW‐ Wisconsin Alzheimer's Institute, Milwaukee, WI, USA; ^5^ Wisconsin Research and Education Network, University of Wisconsin School of Medicine and Public Health, Madison, WI, USA

## Abstract

**Background:**

The majority of persons living with dementia (PLWD) experience at least one behavioral and psychological symptoms of dementia (BPSD) during their illness.^1,2^ Unmanaged BPSD can result in increased caregiver stress, hospitalization risk, emergency service use, earlier nursing home placement, and decline in quality of life for both PLWD and their caregivers.^3,4,5,10^ Due to a lack of trained geriatricians, most individuals with BPSD receive care from primary care clinicians (PCPs) who report lacking the knowledge and self‐efficacy to manage these symptoms effectively.^6,7,8^ DETAILD (Dementia Educational Techniques: Academic detaILing and DICE) is an intervention designed to enhance knowledge, confidence, and practice changes for better recognition and management of BPSD in primary care teams.^9,10,11^ Our purpose was to identify the challenges clinicians face with PLWD and their caregivers in managing BPSD and adopting the DETAILD approach.

**Method:**

Guided by the RE‐AIM/PRISM framework^12^, semi‐structured interviews were conducted with 14 clinicians in primary care settings serving underserved communities. Seven practiced in primary care‐integrated memory clinics with extensive DETAILD training, and seven outside those clinics had some to no training. Qualitative analysis was performed on transcribed interview content using inductive and deductive approaches^13^, incorporating both the RE‐AIM framework and multi‐level domains^14^.

**Result:**

Most clinicians interviewed were White (93%), female (65%), physicians (64%), and all were non‐Hispanic or Latino. Clinicians practiced in Family Medicine, Internal Medicine and Behavioral Health, in various urban and rural settings. Several themes emerged regardless of DETAILD experience: (1) Need for care planning communication, coordination and collaboration, (2) Need for family and caregiver support/buy‐in, (3) Impact of family and household dynamics, and (4) Benefits of patient/family participation in case consultations. Themes identified as barriers only by clinicians with no DETAILD experience include 5) Lack of a diagnosis and (6) Impact of patient trust and understanding on case consultations.

**Conclusion:**

This study highlights the barriers PCPs encounter when consulting with patients and their caregivers to manage BPSD. The findings will inform adaptations of DETAILD to increase participation and its impact on practice change, ultimately improving dementia care in primary care settings and enhancing the quality of life for PLWD and their caregivers.

